# (3R*,4R*,5S*)-4-(4-Methyl­phen­yl)-2,3-diphenyl-7-[(R*)-1-phenyl­ethyl]-1-oxa-2,7-diaza­spiro­[4.5]decan-10-one oxime

**DOI:** 10.1107/S1600536808004121

**Published:** 2008-02-15

**Authors:** G. Chanthini Begum, R. Suresh Kumar, J. Suresh, C. Gopinathan, P. L. Nilantha Lakshman

**Affiliations:** a Department of Physics, The Madura College, Madurai 625 011, India; b School of Chemistry, Madurai Kamaraj University, Madurai 625 021, India; c Department of Food Science and Technology, Faculty of Agriculture, University of Ruhuna, Mapalana, Kamburupitiya 81100, Sri Lanka

## Abstract

In the title compound, C_34_H_35_N_3_O_2_, the polysubstituted piperidine ring adopts a chair conformation and the isoxazolidine ring is in an envelope form. The mol­ecules are linked into a chain along the *b* axis by O—H⋯N, C—H⋯O and C—H⋯N inter­actions. The chains are cross-linked *via* weak C—H⋯π inter­actions.

## Related literature

For related literature, see: Ali *et al.* (1988 ▶); Annuziata *et al.* (1987 ▶); Colombi *et al.* (1978 ▶); Gothelf & Jorgensen (2000 ▶); Goti *et al.* (1997 ▶); Hossain *et al.* (1993 ▶); Kumar *et al.* (2003 ▶).
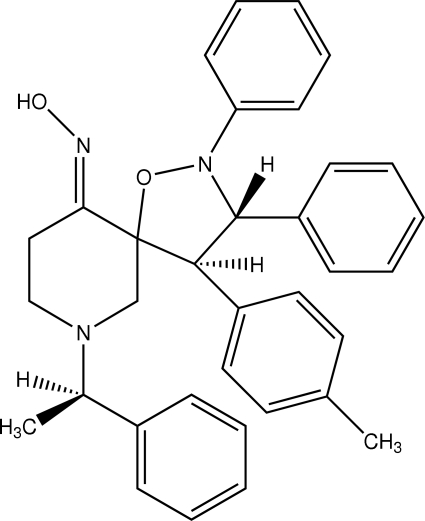


## Experimental

### 

#### Crystal data

C_34_H_35_N_3_O_2_
*M* *_r_* = 517.65
Orthorhombic, 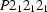

*a* = 10.448 (7) Å
*b* = 10.588 (9) Å
*c* = 26.490 (16) Å
*V* = 2930 (4) Å^3^
*Z* = 4
Mo *K*α radiation
μ = 0.07 mm^−1^
*T* = 293 (2) K
0.18 × 0.16 × 0.11 mm

#### Data collection

Nonius MACH-3 diffractometer
Absorption correction: ψ scan (North *et al.*, 1968 ▶) *T*_min_ = 0.986, *T*_max_ = 0.991
3068 measured reflections
2933 independent reflections
1037 reflections with *I* > 2σ(*I*)
*R*_int_ = 0.049
2 standard reflections frequency: 60 min intensity decay: none

#### Refinement

*R*[*F* ^2^ > 2σ(*F* ^2^)] = 0.050
*wR*(*F* ^2^) = 0.119
*S* = 0.95
2933 reflections
355 parameters
H-atom parameters constrained
Δρ_max_ = 0.15 e Å^−3^
Δρ_min_ = −0.17 e Å^−3^

### 

Data collection: *CAD-4 EXPRESS* (Enraf–Nonius, 1994 ▶); cell refinement: *CAD-4 EXPRESS*; data reduction: *XCAD4* (Harms & Wocadlo, 1996 ▶); program(s) used to solve structure: *SHELXS97* (Sheldrick, 2008 ▶); program(s) used to refine structure: *SHELXL97* (Sheldrick, 2008 ▶); molecular graphics: *PLATON* (Spek, 2003 ▶); software used to prepare material for publication: *SHELXL97*.

## Supplementary Material

Crystal structure: contains datablocks global, I. DOI: 10.1107/S1600536808004121/ci2561sup1.cif

Structure factors: contains datablocks I. DOI: 10.1107/S1600536808004121/ci2561Isup2.hkl

Additional supplementary materials:  crystallographic information; 3D view; checkCIF report

## Figures and Tables

**Table 1:** Hydrogen-bond geometry (Å, °) *Cg*1 is the centroid of the C31–C36 ring.

*D*—H⋯*A*	*D*—H	H⋯*A*	*D*⋯*A*	*D*—H⋯*A*
O1—H1⋯N1^i^	0.82	1.98	2.791 (6)	170
C3—H3*B*⋯N2^ii^	0.97	2.61	3.353 (8)	133
C96—H96⋯O1^ii^	0.93	2.60	3.456 (9)	154
C94—H94⋯*Cg*1^iii^	0.93	2.80	3.721 (11)	170

Symmetry codes: (i) 
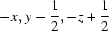
; (ii) 
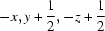
; (iii) 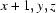.

**Fig. 1.**
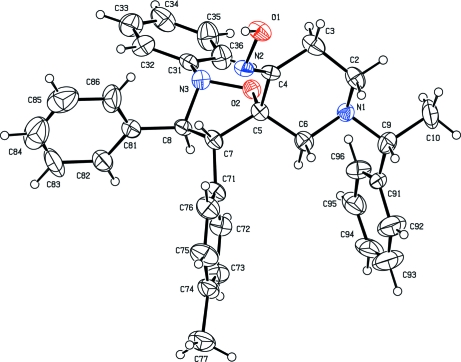
The molecular structure of the title compound, showing 30% probability displacement ellipsoids and the atom-numbering scheme.

## supplementary crystallographic information

### Comment

1,3-dipolar cycloaddition of nitrones with olefinic dipolarophiles proceeds through a concerted mechanism yielding highly substituted isoxazolidines with generation of as many as three new contiguous stereogenic centers in a single step (Gothelf & Jorgensen, 2000). Isoxazolidines are potential precursors for biologically important compounds such as amino sugars, alkaloids (Goti *et al.*, 1997; Ali *et al.*, 1988), β-lactams (Ali *et al.*, 1988), and amino acids (Annuziata *et al.*, 1987), and exhibit antibacterial and antifungal activities (Kumar *et al.*, 2003). Among the dipoles, nitrones have been extensively used as they readily undergo both inter- and intra-molecular 1,3-dipolar cycloaddition with olefins. 1,3-dipolar cycloaddition of exocyclic olefins with nitrones result in highly substituted spiro-isoxazolidines (Hossain *et al.*, 1993) and they have also been transformed into complex heterocycles (Colombi *et al.*, 1978).

The molecular structure of the title compound is shown in Fig.1. The isoxazolidine ring has an envelope conformation, as indicated by the puckering parameters Q = 0.492 (6) Å and φ = 34.1 (7)°. The piperidine ring adopts a chair conformation. The C31—C36, C81—C86 and C71—C76 phenyl rings form dihedral angles of 37.5 (3)°, 77.5 (3)° and 71.8 (2)°, respectively, with the O2/C5/C7/C8 plane. The C31—C36 and C71—C76 phenyl rings are oriented at angles of 74.1 (3)° and 70.9 (3)°, respectively, with respect to the C81—C86 phenyl ring. The C2—N1—C9—C91 and C6—N1—C9—C10 torsion angles are 175.9 (6) and 178.4 (5)°, respectively.

Intermolecular O—H···N and weak C—H···O and C—H···N interactions form a linear chain running parallel to the *b* axis (Table 1). The chains are cross-linked *via* weak C—H···π interactions involving the C31—C36 phenyl ring (centroid *Cg*1).

### Experimental

4-(4-Methylphenyl)-2,3-diphenyl-7-[(*R*)-1-phenylethyl]-1-oxa-2,7-diazaspiro[4.5] decan-10-one (0.05 g, 0.01 mmol), hydroxylammonium chloride (0.010 g, 0.015 mmol) and sodium acetate (0.012 g, 0.015 mmol) in ethanol (3 ml) was refluxed for 30 min. After completion of the reaction, as evident from TLC the excess solvent was evaporated *in vacuo* and the residue was subjected to flash column chromatography on silica gel using petroleum ether-ethyl acetate (10:2) as eluent. The product was recrystallized from ethanol (yield 72%, m.p 418 K)

### Refinement

H atoms were placed at calculated positions and allowed to ride on their carrier atoms with C—H = 0.93–0.98 Å, O—H = 0.82 Å and *U*_iso_ = 1.2*U*_eq_(C) for CH_2_ and CH groups, and 1.5*U*_eq_ for CH_3_ and OH groups. In the absence of significant anomalous scattering, the absolute configuration could not be reliably determined and Friedel pairs were merged.

### Crystal data

**Table:**

C_34_H_35_N_3_O_2_	*F*_000_ = 1104
*M**_r_* = 517.65	*D*_x_ = 1.173 Mg m^−^^3^
Orthorhombic, *P*2_1_2_1_2_1_	Mo *K*α radiation λ = 0.71073 Å
Hall symbol: P 2ac 2ab	Cell parameters from 25 reflections
*a* = 10.448 (7) Å	θ = 2–25º
*b* = 10.588 (9) Å	µ = 0.07 mm^−^^1^
*c* = 26.490 (16) Å	*T* = 293 (2) K
*V* = 2930 (4) Å^3^	Block, colourless
*Z* = 4	0.18 × 0.16 × 0.11 mm

### Data collection

**Table:**

Nonius MACH-3 diffractometer	*R*_int_ = 0.049
Radiation source: fine-focus sealed tube	θ_max_ = 25.0º
Monochromator: graphite	θ_min_ = 2.1º
*T* = 293(2) K	*h* = 0→12
ω–2θ scans	*k* = 0→12
Absorption correction: ψ scan(North *et al.*, 1968)	*l* = −1→31
*T*_min_ = 0.986, *T*_max_ = 0.991	2 standard reflections
3068 measured reflections	every 60 min
2933 independent reflections	intensity decay: none
1037 reflections with *I* > 2σ(*I*)	

### Refinement

**Table:**

Refinement on *F*^2^	Secondary atom site location: difference Fourier map
Least-squares matrix: full	Hydrogen site location: inferred from neighbouring sites
*R*[*F*^2^ > 2σ(*F*^2^)] = 0.050	H-atom parameters constrained
*wR*(*F*^2^) = 0.119	*w* = 1/[σ^2^(*F*_o_^2^) + (0.0339*P*)^2^] where *P* = (*F*_o_^2^ + 2*F*_c_^2^)/3
*S* = 0.96	(Δ/σ)_max_ = 0.001
2933 reflections	Δρ_max_ = 0.15 e Å^−^^3^
355 parameters	Δρ_min_ = −0.17 e Å^−^^3^
Primary atom site location: structure-invariant direct methods	Extinction correction: none

### Special details

**Table:**

Geometry. All e.s.d.'s (except the e.s.d. in the dihedral angle between two l.s. planes) are estimated using the full covariance matrix. The cell e.s.d.'s are taken into account individually in the estimation of e.s.d.'s in distances, angles and torsion angles; correlations between e.s.d.'s in cell parameters are only used when they are defined by crystal symmetry. An approximate (isotropic) treatment of cell e.s.d.'s is used for estimating e.s.d.'s involving l.s. planes.
Refinement. Refinement of *F*^2^ against ALL reflections. The weighted *R*-factor *wR* and goodness of fit *S* are based on *F*^2^, conventional *R*-factors *R* are based on *F*, with *F* set to zero for negative *F*^2^. The threshold expression of *F*^2^ > σ(*F*^2^) is used only for calculating *R*-factors(gt) *etc*. and is not relevant to the choice of reflections for refinement. *R*-factors based on *F*^2^ are statistically about twice as large as those based on *F*, and *R*- factors based on ALL data will be even larger.

### Fractional atomic coordinates and isotropic or equivalent isotropic displacement parameters (Å^2^)

**Table:**

	*x*	*y*	*z*	*U*_iso_*/*U*_eq_	
C2	0.1491 (7)	0.2999 (7)	0.2896 (2)	0.057 (2)	
H2A	0.1502	0.3589	0.3176	0.068*	
H2B	0.1778	0.2185	0.3020	0.068*	
C3	0.0135 (7)	0.2879 (6)	0.2695 (2)	0.061 (2)	
H3A	−0.0421	0.2557	0.2959	0.073*	
H3B	−0.0178	0.3704	0.2594	0.073*	
C4	0.0111 (6)	0.2011 (7)	0.2257 (2)	0.0428 (19)	
C5	0.1068 (6)	0.2330 (6)	0.1843 (2)	0.0440 (19)	
C6	0.2378 (6)	0.2497 (7)	0.2084 (2)	0.0495 (19)	
H6A	0.2663	0.1691	0.2218	0.059*	
H6B	0.2985	0.2757	0.1827	0.059*	
C7	0.1025 (6)	0.1445 (6)	0.1374 (2)	0.0453 (19)	
H7	0.0322	0.0849	0.1430	0.054*	
C8	0.0593 (6)	0.2344 (6)	0.0951 (2)	0.0459 (19)	
H8	0.1348	0.2757	0.0806	0.055*	
C9	0.3678 (7)	0.3610 (7)	0.2705 (3)	0.059 (2)	
H9	0.3949	0.2804	0.2852	0.070*	
C10	0.3704 (8)	0.4613 (7)	0.3123 (2)	0.092 (3)	
H10A	0.3400	0.5402	0.2990	0.138*	
H10B	0.4564	0.4714	0.3243	0.138*	
H10C	0.3163	0.4350	0.3396	0.138*	
C31	−0.0448 (7)	0.4453 (7)	0.0987 (3)	0.049 (2)	
C32	−0.1389 (8)	0.4467 (8)	0.0614 (3)	0.069 (2)	
H32	−0.1766	0.3712	0.0515	0.083*	
C33	−0.1768 (8)	0.5572 (10)	0.0391 (3)	0.081 (3)	
H33	−0.2395	0.5554	0.0142	0.098*	
C34	−0.1238 (10)	0.6699 (8)	0.0530 (3)	0.078 (3)	
H34	−0.1507	0.7451	0.0383	0.094*	
C35	−0.0303 (9)	0.6693 (8)	0.0890 (3)	0.084 (3)	
H35	0.0081	0.7452	0.0980	0.101*	
C36	0.0098 (8)	0.5574 (8)	0.1127 (3)	0.065 (2)	
H36	0.0726	0.5595	0.1376	0.078*	
C71	0.2166 (7)	0.0680 (7)	0.1229 (2)	0.047 (2)	
C72	0.3230 (7)	0.1135 (7)	0.0978 (3)	0.057 (2)	
H72	0.3307	0.1998	0.0921	0.068*	
C73	0.4189 (8)	0.0327 (7)	0.0807 (3)	0.065 (2)	
H73	0.4884	0.0662	0.0633	0.078*	
C74	0.4133 (9)	−0.0953 (8)	0.0889 (3)	0.065 (2)	
C75	0.3100 (9)	−0.1418 (8)	0.1145 (3)	0.081 (3)	
H75	0.3036	−0.2281	0.1207	0.098*	
C76	0.2152 (8)	−0.0616 (8)	0.1312 (3)	0.067 (2)	
H76	0.1467	−0.0960	0.1489	0.081*	
C77	0.5171 (7)	−0.1818 (8)	0.0674 (3)	0.093 (3)	
H77A	0.5320	−0.1608	0.0327	0.139*	
H77B	0.4897	−0.2681	0.0699	0.139*	
H77C	0.5948	−0.1707	0.0863	0.139*	
C81	−0.0138 (8)	0.1710 (7)	0.0537 (3)	0.057 (2)	
C82	0.0338 (8)	0.1661 (7)	0.0054 (3)	0.079 (3)	
H82	0.1112	0.2050	−0.0022	0.094*	
C83	−0.0344 (13)	0.1023 (11)	−0.0326 (4)	0.116 (5)	
H83	−0.0023	0.0992	−0.0654	0.139*	
C84	−0.1453 (13)	0.0462 (13)	−0.0216 (6)	0.136 (6)	
H84	−0.1877	0.0016	−0.0468	0.163*	
C85	−0.1984 (11)	0.0517 (11)	0.0251 (5)	0.120 (4)	
H85	−0.2780	0.0161	0.0316	0.144*	
C86	−0.1287 (10)	0.1135 (8)	0.0636 (3)	0.088 (3)	
H86	−0.1613	0.1151	0.0962	0.105*	
C91	0.4635 (7)	0.3974 (8)	0.2304 (3)	0.056 (2)	
C92	0.5805 (9)	0.3409 (10)	0.2280 (4)	0.105 (4)	
H92	0.5980	0.2731	0.2492	0.126*	
C93	0.6745 (11)	0.3822 (11)	0.1947 (5)	0.125 (5)	
H93	0.7541	0.3430	0.1938	0.150*	
C94	0.6480 (11)	0.4818 (11)	0.1631 (4)	0.107 (4)	
H94	0.7107	0.5117	0.1412	0.128*	
C95	0.5312 (9)	0.5363 (8)	0.1637 (3)	0.081 (3)	
H95	0.5127	0.6016	0.1415	0.097*	
C96	0.4391 (7)	0.4953 (8)	0.1974 (3)	0.067 (2)	
H96	0.3594	0.5343	0.1978	0.081*	
N1	0.2373 (5)	0.3445 (5)	0.2497 (2)	0.0447 (15)	
N2	−0.0569 (5)	0.1010 (5)	0.2211 (2)	0.0442 (15)	
N3	−0.0164 (5)	0.3297 (5)	0.12360 (19)	0.0462 (15)	
O1	−0.1378 (5)	0.0870 (4)	0.26317 (17)	0.0568 (14)	
H1	−0.1726	0.0177	0.2621	0.085*	
O2	0.0736 (4)	0.3573 (4)	0.16451 (15)	0.0469 (12)	

### Atomic displacement parameters (Å^2^)

**Table:**

	*U*^11^	*U*^22^	*U*^33^	*U*^12^	*U*^13^	*U*^23^
C2	0.077 (6)	0.051 (5)	0.042 (4)	−0.012 (5)	0.010 (5)	−0.003 (4)
C3	0.074 (6)	0.051 (5)	0.057 (5)	−0.007 (5)	0.021 (5)	−0.007 (4)
C4	0.041 (5)	0.046 (4)	0.041 (4)	0.004 (4)	0.013 (4)	−0.001 (4)
C5	0.049 (5)	0.042 (5)	0.041 (4)	−0.001 (4)	0.002 (4)	−0.001 (4)
C6	0.058 (5)	0.049 (4)	0.042 (4)	0.000 (4)	0.002 (4)	0.004 (4)
C7	0.058 (5)	0.038 (4)	0.040 (4)	−0.008 (4)	0.007 (4)	−0.003 (4)
C8	0.042 (5)	0.053 (5)	0.043 (4)	0.000 (4)	0.007 (4)	−0.011 (4)
C9	0.056 (6)	0.066 (6)	0.053 (5)	0.005 (5)	−0.013 (5)	0.009 (5)
C10	0.106 (7)	0.120 (7)	0.050 (5)	−0.019 (6)	−0.019 (5)	−0.026 (6)
C31	0.050 (5)	0.049 (5)	0.047 (5)	0.000 (5)	0.012 (4)	−0.011 (5)
C32	0.055 (6)	0.073 (7)	0.080 (6)	0.004 (5)	−0.003 (5)	0.002 (6)
C33	0.085 (7)	0.093 (7)	0.066 (6)	0.027 (7)	−0.008 (5)	0.014 (7)
C34	0.116 (9)	0.058 (7)	0.061 (6)	0.011 (6)	0.012 (6)	−0.001 (5)
C35	0.114 (8)	0.071 (7)	0.068 (6)	−0.005 (6)	−0.020 (6)	0.006 (5)
C36	0.087 (7)	0.053 (5)	0.057 (5)	0.004 (6)	−0.012 (5)	0.011 (5)
C71	0.064 (6)	0.031 (5)	0.047 (5)	−0.004 (5)	0.011 (4)	−0.003 (4)
C72	0.061 (5)	0.051 (5)	0.058 (5)	0.005 (5)	0.008 (5)	0.006 (5)
C73	0.063 (6)	0.068 (6)	0.065 (5)	0.010 (5)	0.015 (5)	0.014 (5)
C74	0.081 (7)	0.064 (6)	0.050 (5)	0.031 (6)	−0.002 (5)	−0.006 (5)
C75	0.107 (8)	0.049 (5)	0.087 (7)	0.016 (6)	0.019 (6)	0.002 (5)
C76	0.078 (6)	0.065 (6)	0.059 (6)	−0.004 (6)	0.012 (5)	0.005 (5)
C77	0.100 (7)	0.090 (7)	0.088 (6)	0.042 (6)	0.008 (6)	−0.017 (5)
C81	0.057 (6)	0.061 (5)	0.052 (5)	0.012 (5)	−0.002 (5)	−0.011 (5)
C82	0.086 (6)	0.089 (6)	0.060 (5)	0.029 (6)	−0.011 (5)	−0.021 (5)
C83	0.174 (13)	0.117 (11)	0.056 (6)	0.065 (10)	−0.037 (8)	−0.044 (7)
C84	0.132 (14)	0.090 (10)	0.187 (15)	0.019 (10)	−0.070 (13)	−0.049 (11)
C85	0.089 (9)	0.085 (8)	0.186 (13)	0.013 (7)	−0.012 (10)	−0.017 (10)
C86	0.094 (8)	0.076 (7)	0.093 (8)	−0.010 (6)	−0.033 (7)	−0.028 (6)
C91	0.037 (5)	0.066 (6)	0.064 (5)	0.013 (5)	−0.011 (4)	−0.025 (5)
C92	0.062 (7)	0.110 (8)	0.144 (10)	0.024 (7)	−0.005 (7)	−0.021 (8)
C93	0.056 (7)	0.138 (12)	0.181 (14)	0.028 (9)	0.000 (8)	−0.036 (10)
C94	0.064 (8)	0.135 (11)	0.122 (10)	−0.025 (8)	0.048 (7)	−0.063 (8)
C95	0.083 (7)	0.090 (7)	0.070 (5)	−0.012 (7)	0.021 (6)	−0.016 (5)
C96	0.048 (5)	0.073 (6)	0.082 (6)	0.001 (5)	0.017 (5)	0.003 (5)
N1	0.044 (4)	0.043 (3)	0.047 (3)	−0.001 (3)	−0.004 (3)	−0.004 (3)
N2	0.044 (4)	0.042 (3)	0.046 (4)	0.000 (3)	0.011 (3)	−0.002 (3)
N3	0.052 (4)	0.042 (4)	0.044 (3)	−0.003 (3)	0.000 (3)	0.005 (3)
O1	0.062 (4)	0.053 (4)	0.056 (3)	−0.015 (3)	0.024 (3)	−0.001 (3)
O2	0.052 (3)	0.047 (3)	0.042 (3)	−0.001 (3)	0.001 (3)	0.002 (3)

### Geometric parameters (Å, °)

**Table:**

C2—N1	1.480 (7)	C71—C72	1.383 (8)
C2—C3	1.518 (8)	C71—C76	1.390 (8)
C2—H2A	0.97	C72—C73	1.393 (9)
C2—H2B	0.97	C72—H72	0.93
C3—C4	1.482 (8)	C73—C74	1.374 (9)
C3—H3A	0.97	C73—H73	0.93
C3—H3B	0.97	C74—C75	1.367 (10)
C4—N2	1.282 (7)	C74—C77	1.529 (9)
C4—C5	1.520 (8)	C75—C76	1.377 (10)
C5—O2	1.460 (7)	C75—H75	0.93
C5—C6	1.521 (8)	C76—H76	0.93
C5—C7	1.557 (8)	C77—H77A	0.96
C6—N1	1.485 (7)	C77—H77B	0.96
C6—H6A	0.97	C77—H77C	0.96
C6—H6B	0.97	C81—C86	1.371 (10)
C7—C71	1.491 (8)	C81—C82	1.374 (8)
C7—C8	1.537 (8)	C82—C83	1.406 (11)
C7—H7	0.98	C82—H82	0.93
C8—N3	1.487 (7)	C83—C84	1.335 (14)
C8—C81	1.496 (9)	C83—H83	0.93
C8—H8	0.98	C84—C85	1.358 (15)
C9—N1	1.481 (7)	C84—H84	0.93
C9—C91	1.508 (9)	C85—C86	1.412 (12)
C9—C10	1.534 (8)	C85—H85	0.93
C9—H9	0.98	C86—H86	0.93
C10—H10A	0.96	C91—C92	1.362 (10)
C10—H10B	0.96	C91—C96	1.379 (9)
C10—H10C	0.96	C92—C93	1.391 (13)
C31—C36	1.368 (8)	C92—H92	0.93
C31—C32	1.393 (9)	C93—C94	1.374 (13)
C31—N3	1.422 (8)	C93—H93	0.93
C32—C33	1.369 (10)	C94—C95	1.350 (11)
C32—H32	0.93	C94—H94	0.93
C33—C34	1.366 (10)	C95—C96	1.382 (9)
C33—H33	0.93	C95—H95	0.93
C34—C35	1.365 (10)	C96—H96	0.93
C34—H34	0.93	N2—O1	1.406 (6)
C35—C36	1.405 (10)	N3—O2	1.464 (6)
C35—H35	0.93	O1—H1	0.82
C36—H36	0.93		
			
N1—C2—C3	111.0 (5)	C72—C71—C76	115.4 (7)
N1—C2—H2A	109.4	C72—C71—C7	125.2 (6)
C3—C2—H2A	109.4	C76—C71—C7	119.2 (7)
N1—C2—H2B	109.4	C71—C72—C73	121.4 (7)
C3—C2—H2B	109.4	C71—C72—H72	119.3
H2A—C2—H2B	108.0	C73—C72—H72	119.3
C4—C3—C2	110.0 (6)	C74—C73—C72	121.6 (8)
C4—C3—H3A	109.7	C74—C73—H73	119.2
C2—C3—H3A	109.7	C72—C73—H73	119.2
C4—C3—H3B	109.7	C75—C74—C73	117.9 (8)
C2—C3—H3B	109.7	C75—C74—C77	121.9 (8)
H3A—C3—H3B	108.2	C73—C74—C77	120.1 (9)
N2—C4—C3	126.6 (6)	C74—C75—C76	120.3 (8)
N2—C4—C5	118.7 (6)	C74—C75—H75	119.8
C3—C4—C5	114.6 (6)	C76—C75—H75	119.8
O2—C5—C4	107.6 (5)	C75—C76—C71	123.4 (8)
O2—C5—C6	105.1 (5)	C75—C76—H76	118.3
C4—C5—C6	108.4 (6)	C71—C76—H76	118.3
O2—C5—C7	104.4 (5)	C74—C77—H77A	109.5
C4—C5—C7	115.0 (5)	C74—C77—H77B	109.5
C6—C5—C7	115.5 (6)	H77A—C77—H77B	109.5
N1—C6—C5	112.6 (5)	C74—C77—H77C	109.5
N1—C6—H6A	109.1	H77A—C77—H77C	109.5
C5—C6—H6A	109.1	H77B—C77—H77C	109.5
N1—C6—H6B	109.1	C86—C81—C82	118.5 (8)
C5—C6—H6B	109.1	C86—C81—C8	120.5 (7)
H6A—C6—H6B	107.8	C82—C81—C8	121.0 (8)
C71—C7—C8	112.6 (5)	C81—C82—C83	120.1 (9)
C71—C7—C5	120.6 (6)	C81—C82—H82	119.9
C8—C7—C5	102.6 (5)	C83—C82—H82	119.9
C71—C7—H7	106.7	C84—C83—C82	119.8 (12)
C8—C7—H7	106.7	C84—C83—H83	120.1
C5—C7—H7	106.7	C82—C83—H83	120.1
N3—C8—C81	113.9 (6)	C83—C84—C85	122.4 (15)
N3—C8—C7	101.9 (5)	C83—C84—H84	118.8
C81—C8—C7	114.0 (5)	C85—C84—H84	118.8
N3—C8—H8	108.9	C84—C85—C86	117.8 (13)
C81—C8—H8	108.9	C84—C85—H85	121.1
C7—C8—H8	108.9	C86—C85—H85	121.1
N1—C9—C91	112.2 (6)	C81—C86—C85	121.3 (10)
N1—C9—C10	111.5 (6)	C81—C86—H86	119.3
C91—C9—C10	108.6 (6)	C85—C86—H86	119.3
N1—C9—H9	108.1	C92—C91—C96	117.8 (9)
C91—C9—H9	108.1	C92—C91—C9	121.1 (9)
C10—C9—H9	108.1	C96—C91—C9	121.0 (7)
C9—C10—H10A	109.5	C91—C92—C93	121.7 (11)
C9—C10—H10B	109.5	C91—C92—H92	119.1
H10A—C10—H10B	109.5	C93—C92—H92	119.1
C9—C10—H10C	109.5	C94—C93—C92	119.0 (12)
H10A—C10—H10C	109.5	C94—C93—H93	120.5
H10B—C10—H10C	109.5	C92—C93—H93	120.5
C36—C31—C32	118.5 (7)	C95—C94—C93	120.1 (12)
C36—C31—N3	122.3 (7)	C95—C94—H94	119.9
C32—C31—N3	119.1 (7)	C93—C94—H94	119.9
C33—C32—C31	121.3 (8)	C94—C95—C96	120.3 (9)
C33—C32—H32	119.4	C94—C95—H95	119.9
C31—C32—H32	119.4	C96—C95—H95	119.9
C34—C33—C32	120.9 (8)	C91—C96—C95	121.0 (8)
C34—C33—H33	119.5	C91—C96—H96	119.5
C32—C33—H33	119.5	C95—C96—H96	119.5
C35—C34—C33	118.3 (9)	C2—N1—C9	110.2 (5)
C35—C34—H34	120.9	C2—N1—C6	108.2 (5)
C33—C34—H34	120.9	C9—N1—C6	110.5 (5)
C34—C35—C36	122.0 (9)	C4—N2—O1	110.3 (5)
C34—C35—H35	119.0	C31—N3—O2	107.8 (5)
C36—C35—H35	119.0	C31—N3—C8	117.4 (5)
C31—C36—C35	119.1 (7)	O2—N3—C8	99.8 (5)
C31—C36—H36	120.5	N2—O1—H1	109.5
C35—C36—H36	120.5	C5—O2—N3	103.8 (4)
			
N1—C2—C3—C4	57.3 (8)	N3—C8—C81—C82	128.0 (7)
C2—C3—C4—N2	123.3 (8)	C7—C8—C81—C82	−115.7 (8)
C2—C3—C4—C5	−52.3 (7)	C86—C81—C82—C83	−0.5 (12)
N2—C4—C5—O2	121.4 (6)	C8—C81—C82—C83	177.8 (7)
C3—C4—C5—O2	−62.5 (7)	C81—C82—C83—C84	−0.2 (16)
N2—C4—C5—C6	−125.4 (6)	C82—C83—C84—C85	2(2)
C3—C4—C5—C6	50.6 (8)	C83—C84—C85—C86	−4(2)
N2—C4—C5—C7	5.6 (9)	C82—C81—C86—C85	−0.9 (13)
C3—C4—C5—C7	−178.4 (5)	C8—C81—C86—C85	−179.2 (8)
O2—C5—C6—N1	60.0 (6)	C84—C85—C86—C81	2.9 (16)
C4—C5—C6—N1	−54.9 (7)	N1—C9—C91—C92	−135.1 (7)
C7—C5—C6—N1	174.4 (5)	C10—C9—C91—C92	101.2 (9)
O2—C5—C7—C71	123.6 (6)	N1—C9—C91—C96	49.8 (9)
C4—C5—C7—C71	−118.7 (7)	C10—C9—C91—C96	−73.9 (8)
C6—C5—C7—C71	8.8 (9)	C96—C91—C92—C93	2.0 (13)
O2—C5—C7—C8	−2.5 (7)	C9—C91—C92—C93	−173.3 (9)
C4—C5—C7—C8	115.2 (6)	C91—C92—C93—C94	−0.6 (17)
C6—C5—C7—C8	−117.3 (6)	C92—C93—C94—C95	−1.6 (17)
C71—C7—C8—N3	−159.2 (5)	C93—C94—C95—C96	2.3 (15)
C5—C7—C8—N3	−28.1 (6)	C92—C91—C96—C95	−1.2 (11)
C71—C7—C8—C81	77.7 (7)	C9—C91—C96—C95	174.0 (7)
C5—C7—C8—C81	−151.2 (6)	C94—C95—C96—C91	−0.9 (12)
C36—C31—C32—C33	−0.2 (11)	C3—C2—N1—C9	177.5 (6)
N3—C31—C32—C33	174.9 (7)	C3—C2—N1—C6	−61.6 (7)
C31—C32—C33—C34	−0.3 (13)	C91—C9—N1—C2	175.9 (6)
C32—C33—C34—C35	1.2 (13)	C10—C9—N1—C2	−62.0 (7)
C33—C34—C35—C36	−1.7 (13)	C91—C9—N1—C6	56.3 (7)
C32—C31—C36—C35	−0.3 (11)	C10—C9—N1—C6	178.4 (5)
N3—C31—C36—C35	−175.1 (7)	C5—C6—N1—C2	61.5 (7)
C34—C35—C36—C31	1.2 (13)	C5—C6—N1—C9	−177.8 (6)
C8—C7—C71—C72	42.9 (9)	C3—C4—N2—O1	3.8 (9)
C5—C7—C71—C72	−78.4 (9)	C5—C4—N2—O1	179.3 (5)
C8—C7—C71—C76	−131.6 (7)	C36—C31—N3—O2	1.4 (8)
C5—C7—C71—C76	107.0 (8)	C32—C31—N3—O2	−173.4 (6)
C76—C71—C72—C73	2.1 (11)	C36—C31—N3—C8	−110.1 (7)
C7—C71—C72—C73	−172.6 (7)	C32—C31—N3—C8	75.0 (8)
C71—C72—C73—C74	−1.2 (12)	C81—C8—N3—C31	−72.3 (8)
C72—C73—C74—C75	0.1 (13)	C7—C8—N3—C31	164.5 (6)
C72—C73—C74—C77	177.1 (6)	C81—C8—N3—O2	171.7 (5)
C73—C74—C75—C76	0.1 (13)	C7—C8—N3—O2	48.5 (5)
C77—C74—C75—C76	−176.8 (7)	C4—C5—O2—N3	−89.7 (5)
C74—C75—C76—C71	0.9 (13)	C6—C5—O2—N3	154.9 (4)
C72—C71—C76—C75	−1.9 (12)	C7—C5—O2—N3	33.0 (6)
C7—C71—C76—C75	173.1 (7)	C31—N3—O2—C5	−174.5 (5)
N3—C8—C81—C86	−53.8 (9)	C8—N3—O2—C5	−51.4 (5)
C7—C8—C81—C86	62.6 (9)		

### Hydrogen-bond geometry (Å, °)

**Table:**

*D*—H···*A*	*D*—H	H···*A*	*D*···*A*	*D*—H···*A*
O1—H1···N1^i^	0.82	1.98	2.791 (6)	170
C3—H3B···N2^ii^	0.97	2.61	3.353 (8)	133
C96—H96···O1^ii^	0.93	2.60	3.456 (9)	154
C94—H94···Cg1^iii^	0.93	2.80	3.721 (11)	170

Symmetry codes: (i) −*x*, *y*−1/2, −*z*+1/2; (ii) −*x*, *y*+1/2, −*z*+1/2; (iii) *x*+1, *y*, *z*.
